# Molecular detection, quantification, and isolation of *Streptococcus gallolyticus *bacteria colonizing colorectal tumors: inflammation-driven potential of carcinogenesis via IL-1, COX-2, and IL-8

**DOI:** 10.1186/1476-4598-9-249

**Published:** 2010-09-17

**Authors:** Ahmed S Abdulamir, Rand R Hafidh, Fatimah Abu Bakar

**Affiliations:** 1 Institute of Bioscience, University Putra Malaysia, 43400 Serdang, Selangor, Malaysia; 2 Alnahrain University, College of Medicine, 14222, Baghdad, Iraq

## Abstract

**Background:**

Colorectal cancer (CRC) has long been associated with bacteremia and/or endocarditis by *Streptococcus gallolyticus *member bacteria (SGMB) but the direct colonization of SGMB along with its molecular carcinogenic role, if any, has not been investigated. We assessed the colonization of SGMB in CRC patients with history of bacteremia (CRC-w/bac) and without history of bacteremia (CRC-wo/bac) by isolating SGMB from feces, mucosal surfaces of colorectum, and colorectal tissues and detecting SGMB DNA, via PCR and in situ hybridization (ISH) assays targeting *SodA *gene in colorectal tissues. Moreover, mRNA of IL1, IL-8, COX-2, IFN-γ, c-Myc, and Bcl-2 in colorectal tissues of studied groups was assessed via ISH and RT-PCR.

**Results:**

SGMB were found to be remarkably isolated in tumorous (TU) and non-tumorous (NTU) tissues of CRC-w/bac, 20.5% and 17.3%, and CRC-wo/bac, 12.8% and 11.5%, respectively while only 2% of control tissues revealed SGMB (P < 0.05); such contrast was not found in mucosal and fecal isolation of SGMB. The positive detection of SGMB DNA in TU and NTU of CRC-w/bac and CRC-wo/bac via PCR, 48.7%, 35.9%, 32.7%, and 23%, respectively, and ISH, 46.1%, 30.7%, 28.8%, and 17.3%, respectively, was higher than in control tissues, 4 and 2%, respectively (P < 0.05). SGMB count measured via quantitative PCR of SGMB DNA in terms of copy number (CN), in TU and NTU of CRC-w/bac and CRC-wo/bac, 2.96-4.72, 1.29-2.81, 2.16-2.92, and 0.67-2.07 log_10 _CN/g respectively, showed higher colonization in TU than in NTU and in CRC-w/bac than in CRC-wo/bac (P < 0.05). The PCR-based mRNA ratio and ISH-based percentage of positively stained cells of IL-1, 1.77 and 70.3%, COX-2, 1.63 and 44.8%, and IL-8, 1.73 and 70.3%, respectively, rather than IFN-γ, c-Myc, and Bcl-2, were higher in SGMB positive patients than in control or SGMB negative patients (P < 0.05).

**Conclusions:**

The current study indicated that colorectal cancer is remarkably associated with SGMB; moreover, molecular detection of SGMB in CRC was superior to link SGMB with CRC tumors highlighting a possible direct and active role of SGMB in CRC development through most probably inflammation-based sequel of tumor development or propagation via, but not limited to, IL-1, COX-2, and IL-8.

## Background

Colorectal cancer (CRC) is the 4^th ^most common cancer worldwide [1]. Microorganisms were found to be either etiological agents or play a prominent role in the etiology of many types of cancer [2,3]. It has been shown that bacterial infections are possibly linked to cancer by two mechanisms: inflammation and/or formation of carcinogenic metabolites [4]. Therefore, it might be possible to prevent or treat cancer when the infectious source can be identified [5].

One of the bacterial agents associated with cancer is *Streptococcus bovis *(*S. bovis*). *S. bovis *has been found to be important in human health as 25 to 80% of patients with *S. bovis *bacteremia have also a colorectal tumor and the association of colonic neoplasia with *S. bovis *endocarditis has been shown to be 18 to 62% [6-9]. It was shown that 94% of *S. bovis *bacteremia with colorectal cancer is associated with *S. bovis *biotype I while only 18% is associated with biotype II [10]. Later, Osawa et al in 1995 [11] proposed a new species resembling *S. bovis *named *S. gallolyticus*. Interestingly, it was then found that *S. bovis *biotype I and II/2 isolates are in fact *Streptococcus gallolyticus *(*S. gallolyticus*) [12]. Accordingly, *S. bovis *biotype I was replaced by *S. gallolyticus *subspecies *gallolyticus *and biotype II/2 was replaced by *S. gallolyticus *subspecies *pasterianus *and *S. gallolyticus *subspecies *macedonicus *[13]. In the current study, these three taxa were referred to as *S. gallolyticus *member bacteria (SGMB) which have been found to be constantly associated with underlying CRC [12].

Several studies conducted in Asia [14-16] found that *S. gallolyticus *subspecies *gallolyticus *(S. bovis biotype I) and *S. gallolyticus *subspecies *pasterianus *(S. bovis biotype II/2) are the main bacteria associated with colon cancer in Asia. On the other hand, new studies conducted in Germany [17] and Spain [18] found a remarkable association between *S. infantiarus coli *(S. bovis II/1) and colon cancer. Despite the geographical variation, *S. gallolyticus *subspecies *gallolyticus *remains the main bacterium associated with colon cancer worldwide.

No studies were conducted to assess the colonization of SGMB in the colon by detecting SGMB DNA directly in CRC tumors using advanced molecular assays. Therefore, in the current study, SGMB-specific primers and probes in PCR and in situ hybridization (ISH) assays, respectively, together with the bacteriological isolation of SGMB were pursued to detect/isolate SGMB DNA/cells from feces, tumors' mucosal surfaces, and tumors' tissues.

Besides, the nature of SGMB association with CRC was studied in this study since this association has not yet been disclosed clearly. Very few studies [16,19,20] were found to elucidate the underlying mechanism of SGMB association with CRC. Some studies proposed that this association is attributed to the access of SGMB to the circulation via disrupted blood vessels in tumor lesions [19]. However, the appearance of new colonic lesions two to four years after the incidence of SGMB bacteremia/endocarditis [21] and the association of SGMB with preneoplsatic adenomas [16] provided evidence that SGMB might not be a consequence of the tumor lesion. Therefore, in an attempt to clarify the possible role of SGMB colonizing colorectal tissues in triggering, promoting, or propagating CRC, we selected six key factors that might be linked to SGMB colonization and CRC development. Accordingly, two key products of NFkB, namely, interleukin (IL) -1 and COX-2, a central immunological regulator of inflammation, IFN-γ, a central oncogenic factor of CRC, c-Myc, an important antiapoptotic factor, Bcl-2, and a potent angiogenic factor, IL-8, were involved. It is well known that the chronic inflammatory reaction in colorectal mucosa has strongly been implicated in the development of CRC and gastric cancer [19,20]. And it is also known that 70% of CRC is associated with overexpression of c-Myc [22] and most CRC cases are associated with increased antiapoptotic action via increased Bcl-2 expression [23]. And SGMB involvement in CRC patients were found to be strongly associated with increased mRNA expression of IL-8 and NFkB in CRC tumors [16].

## Results

### Demographic and histopathological features of CRC

The demographic and histopathological features of CRC patients are shown in Table 1. The age and sex ratio of CRC patients were found to be not associated with the previous incidence of SGMB bacteremia, namely CRC-wo/bac versus CRC-w/bac groups (*P *= 0.68 and 0.52, respecitvley). In addition, CRC staging and tumor grading were also not associated with previous incidence of SGMB bacteremia, namely CRC-w/bac versus CRC-wo/bac groups (*P *= 0.47 and 0.21, respectively).

**Table 1:** Demographic and histological characteristics of control subjects and colorectal cancer patients

Controls	
Mean age (years)	57.4 ± 4.7
Male: female ratio	1.18: 1
**CRC-wo/bac patients (52 patients)**	

Mean age (years)	59.22 ± 8.18
Male: female ratio	1.2: 1
Duke's staging N (%)	B1: 7 (13.4)
	B2: 7 (13.4)
	C1: 9 (17.3)
	C2: 12 (23)
	D: 17 (32.6)
Tumor differentiation N (%)	Grades 1-2: 21 (40.3)
	Grades 3-4: 31 (59.6)
**CRC-w/bac patients (39 patients)**	

Mean age (years)	56.6 ± 6.7
Male: female ratio	1.16: 1
Duke's staging -N (%)	B1: 4 (10.2)
	B2: 6 (15.3)
	C1: 7 (17.9)
	C2: 10 (25.6)
	D: 13 (33.3)
Tumor differentiation N (%)	Grades 1-2: 17 (43.5)
	Grades 3-4: 22 (56.4)
**Localization of tumors in CRC-w/bac and CRC-wo/bac patients (91 patients)**	

Left colon & rectum -N (%)	48/91 (52.7)
Rectum only - N(%)	15/48 (31.2)
Right colon -N (%)	31/91 (34)
Transverse colon -N (%)	12/91 (13.2)

### Isolation of SGMB

The positive detection/isolation of SGMB and *S. bovis *was found to be mostly impossible without using enrichment media. The positive detection of fecal SGMB was within the normal range, 2.5 to 15% [24]. The frequency of SGMB isolation from feces and mucosal surfaces of normal tissues in control population was not different (*P *= 0.4). Most importantly, the frequency of isolation of SGMB from feces and mucosal surfaces of TU and NTU lesions in both CRC-wo/bac, and CRC-w/bac groups was not different from the corresponding control groups (*P *= 0.77, 0.42, 0.9, 0.71, 0.8, 0.8, respectively) (Table 2).

**Table 2:** Bacteriological isolation of SGMB and *S. bovis *from feces, mucosal surfaces and tissues of colorectum in control, CRC-wo/bac, and CRC-w/bac groups.

Group	Positive isolation of fecal SGMB N (%)	Positive isolation of mucosal SGMB N (%)		Positive isolation of tissue SGMB N (%)	
		**TU**	**NTU**	**TU**	**NTU**

***S. gallolyticus***					
Control (N = 50)	4 (8)	2 (4)		1(2)	
CRC-wo/bac (N = 52)	5 (9.6)	4 (7.6)	2 (3.8)	9 (17.3)	6 (11.5)
CRC-w/bac (N = 39)	4 (10.2)	2 (5.1)	2 (5.1)	8 (20.5)	5 (12.8)
***S. bovis***					
Control (N = 50)	2 (4)	1 (2)		0 (0)	
CRC-wo/bac (N = 52)	2 (3.8)	1 (1.9)	1 (1.9)	0 (0)	0 (0)
CRC-w/bac (N = 39)	1 (2.5)	0 (0)	0 (0)	0 (0)	0 (0)

**Figure 1:**
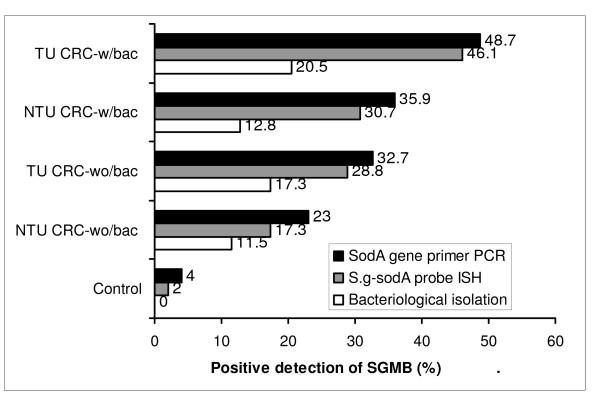
**A histogram showing a comparison in the percentage of the positive detection/isolation of SGMB among control, TU and NTU tissues of CRC-wo/bac, and TU and NTU tissues of CRC-w/bac groups using three detection methods, namely *SodA *gene primer PCR, S.g-*sodA*-probe ISH assay, and enrichment-based bacteriological methods**. It is shown that the top SGMB detection method was PCR, then ISH assay, and least effective was bacteriological isolation.

Unlike the samples of feces and mucosal surfaces of colorectum, SGMB isolated from colorectal tissues were much higher in CRC patients than in control group. High percentage of positive isolation of SGMB was associated with TU (20.5%) and NTU (12.8%) of CRC-w/bac, and TU (17.3%) and NTU (11.5%) of CRC-wo/bac while very low percentage of positive SGMB isolation (2%) was associated with control group (*P *= 0.0008, 0.013, 0.0027, 0.026, respectively) (Table 2, Figure 1). TU specimens showed close figures of positive isolation of SGMB to that in NTU in both CRC-w/bac (*P *= 0.18) and CRC-wo/bac groups (*P *= 0.24). It is worth mentioning that all CRC patients, whose fecal and mucosal surface samples revealed positive isolation of SGMB, showed also positive isolation of SGMB from colorectal tumor tissues but the opposite was not true.

Regarding *S. bovis*, the percentage of its positive isolation was minimal in feces and on the surfaces of colorectal mucosa while it was totally absent in colorectal tissues in all groups (Table 2). The findings of API 20 Strep and Rapid ID32 Strep galleries confirmed the results obtained by the bacteriological tests. All the isolates diagnosed bacteriologically as *S. gallolyticus *from feces, mucosal surfaces, and colorectal tissues were 88, 90.2, and 94.5%, respectively, of biotype I whereas the rest of isolates were shown to be of biotype II/2. On the other hand, all the isolates diagnosed as *S. bovis *were shown to be of biotype II/1. Hence, the bacteriological findings indicated that SGMB rather than *S. bovis *are mainly associated with human bowel and colorectal tissues. And SGMB were within normal percentages in feces or on mucosal surfaces of CRC patients and control group. Nevertheless, SGMB in tumor lesions and safe margins were remarkably found at high levels.

### PCR-based detection of SGMB DNA in colorectal tissues

**Figure 2:**
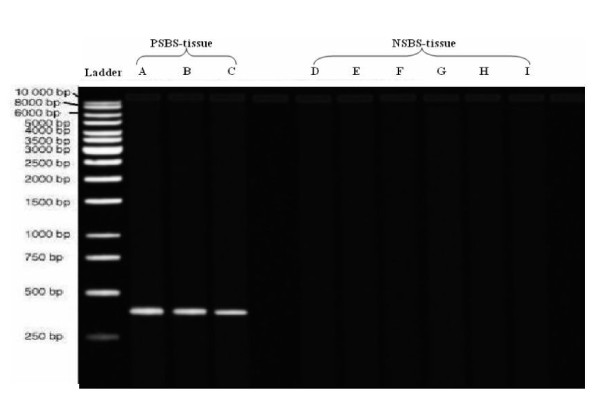
**Gel electrogram of *SodA *gene-specific primer-based PCR products of normal colorectal tissues experimentally inoculated with three PSBS strains (PSBS-tissue) and six NSBS strains (NSBS-tissue)**. PSBS-tissues (lanes A-C) were CIP 105428, CIP 105683, and CIP 105070, respectively while NSBS-tissues (lanes D-I) were CIP 108103, CIP 56.41, CIP 102504, ACM 3539, CIP 76117, and ATCC 25285, respectively. PSBS-tissues, but not NSBS-tissues samples, yielded single band at 408 bp. The used ladder was QIAGEN GelPilot DNA Molecular Weight Marker.

By gel electrophoresis, it was found that a single band of 408 bp PCR product was visible in all PSBS and PSBS-tissue samples while it was absent in all NSBS and NSBS-tissue samples (Figure 2). These findings confirmed the specificity of *SodA *gene primers for the detection of SGMB DNA mixed with larger quantities of DNA of human tissues or DNA of other bacteria found in human bowel.

Since bacteriological methods revealed significant colonization of SGMB in tumor tissues, but not in feces or on mucosal surfaces, PCR-based detection of SGMB DNA was pursued for colorectal tissues. PCR assay revealed more remarkable results than that of bacteriological assays. It was found that the positive detection of SGMB DNA in CRC-wo/bac TU (32.7%) and NTU (23%) tissues was higher than in colorectal tissues of control group (4%) (*P *= 0.0005, p = 0.012, respectively) (Table 3, Figure 1). More strikingly, the positive detection of SGMB DNA in CRC-w/bac TU (48.7%) and NTU (35.9%) tissues was far higher than in colorectal tissues of control group (4%) (*P *= < 0.0001, 0.0003, respectively) (Table 3, Figure 1). There was no single case where NTU was positive for SGMB DNA while the corresponding TU was negative. However, the frequency of positively detected SGMB DNA in TU versus NTU in both CRC-wo/bac and CRC-w/bac groups was not different (*P *= 0.22, 0.25, respectively). Moreover, no significant association was found of the frequency of positively detected SGMB DNA, in both TU and NTU, between CRC-wo/bac versus CRC-w/bac (*P *= 0.12, 0.18, respectively) (Table 3, Figure 1). It is worth mentioning that the positive detection of SGMB by PCR was not associated with stage, grade, and location of tumors (*P *= 0.13, 0.29, 0.48, respectively) or age and sex of patients (*P *= 0.41, 0.22, respectively).

**Table 3:** Detection of SGMB DNA using PCR assay of *SodA *gene-specific primer in colorectal tissues of control, CRC-wo/bac, and CRC-w/bac groups.

Control (N = 50) N (%)	CRC-wo/bac (N = 52) N (%)		CRC-w/bac (N = 39) N (%)	
	**TU**	**NTU**	**TU**	**NTU**
2 (4)	17 (32.7)	12 (23)	19 (48.7)	14 (35.9)

### ISH-based detection of SGMB DNA in colorectal tissues

The positive detection of SGMB DNA via ISH assay was achieved by visualizing SGMB as stained chains of tiny spots scattered in tissue sections. The positive staining was rarely seen as aggregations of stained spots or individually scattered spots. S.g-*sodA *probe of ISH assay yielded positive results in all PSBS-tissue sections, in duplicates, while all the duplicates of NSBS-tissue sections were shown to be negative. Hence, these results provided evidence on the high specificity of S.g-*sodA *probe.

No characteristic localization or distribution of the stained SGMB DNA was found throughout the tested sections. Instead, stained chains of tiny spots were shown to be scattered in tissue sections. SGMB DNA staining in CRC-wo/bac TU (28.8%) and NTU (17.3%) tissues was higher than in colorectal tissues of control group (2%) (*P *= 0.0001, 0.015, respectively) (Table 4, Figure 1) and it was far higher in CRC-w/bac TU (46.1%) and NTU (30.7%) tissues than in colorectal tissues of control group (2%) (*P *= < 0.0001, 0.0001, respectively) (Table 4, Figure 1). There was no single case where NTU was positive for SGMB DNA while the corresponding TU was negative. However, the frequency of positive staining of SGMB DNA in TU versus NTU in both CRC-wo/bac and CRC-w/bac groups was not different (*P *= 0.16, 0.16, respectively) and no significant association was found in the positive staining of SGMB DNA, in TU and NTU, between CRC-wo/bac versus CRC-w/bac groups (*P *= 0.09, 0.13, respectively) (Table 4, Figure 1). Since *SodA *gene primers have been shown to be highly specific for SGMB, it was considered as a golden standard for S.g-*sodA *probe- based ISH assay. Accordingly, the sensitivity and specificity of S.g-*sodA *probe- based ISH assay was 91.6 and 100%, respectively, for TU tissue sections and 87 and 100%, respectively, for NTU tissue sections. Hence, S.g-*sodA *probe has been shown to be as specific as PCR assay but with lower sensitivity. Like PCR, the positive detection of SGMB by ISH was not associated with stage, grade, and location of tumors (*P *= 0.25, 0.11, 0.38, respectively) or age and sex of patients (*P *= 0.26, 0.14, respectively).

**Table 4:** Detection of SGMB DNA using S.g-*sodA *probe-based ISH assay in colorectal tissue sections of control, CRC-wo/bac, and CRC-w/bac groups.

Control (N = 50) N (%)	CRC-wo/bac (N = 52) N (%)		CRC-w/bac (N = 39) N (%)	
	**TU**	**NTU**	**TU**	**NTU**

1 (2)	15 (28.8)	9 (17.3)	18 (46.1)	12 (30.7)

### Quantification of SGMB DNA in colorectal tissues

Based on the positive detection of SGMB DNA, the patients and controls of the current study were categorized into two groups, SGMB+ve and SGMB-ve. For SGMB+ve group, quantitative evaluation of SGMB colonization was sought. The number of bacterial cells, in terms of log_10 _CN/g, was compared among colorectal tissues of the studied groups. First of all, the quantitative real-time PCR confirmed the findings of the conventional PCR without any variation. Above all, the quantitative real-time PCR was successful in quantifying SGMB in colorectal tissues of the studied groups. SGMB load in CRC tissues was expressed as 95% CI of mean log_10_CN/g (Table 5). The SGMB load in TU and NTU of SGMB+ve-CRC-w/bac and TU, but not NTU, of SGMB+ve-CRC-wo/bac was higher than that of SGMB+ve-control group (*P *= < 0.000, 0.042, 0.03, 0.07, respectively). And the difference in SGMB load between TU and NTU, in both SGMB+ve-CRC-wo/bac and SGMB+ve-CRC-w/bac groups, was significant (*P *= 0.01, 0.004, respectively). Moreover, SGMB load in TU, but not NTU, in SGMB+ve-CRC-w/bac was higher than that in SGMB+ve-CRC-wo/bac (*P *= 0.012, 0.2, respectively). On the other hand, upon conducting the quantitative real-time PCR, melting curve analysis of the negative first derivative was pursued. It was found that a single peak at the expected melting temperature of PCR product, Tm 84.3°C, was observed while no significant premature peaks were seen indicating that primer dimers were minimal which provides further evidence on the specific detection of SGMB.

**Table 5:** SGMB load in different groups of CRC tissues, SGMB+ve- control, CRC-wo/bac, and CRC-w/bac by using quantitative real-time *SodA *gene-specific PCR.

SGMB+ve- control	SGMB+ve- CRC-wo/bac		SGMB+ve- CRC-w/bac	
95% CI (log_10_CN/g)	95%CI (log_10_CN/g)		95%CI (log_10_CN/g)	
(N = 2)	TU (N = 17)	NTU (N = 12)	TU (N = 19)	NTU (N = 14)
				
0.48-1.44	2.16-2.92	0.67-2.07	2.96-4.72	1.29-2.81

### The mRNA expression of IFN-γ, COX-2, IL-1, IL-8, c-Myc, and Bcl-2

**Figure 3:**
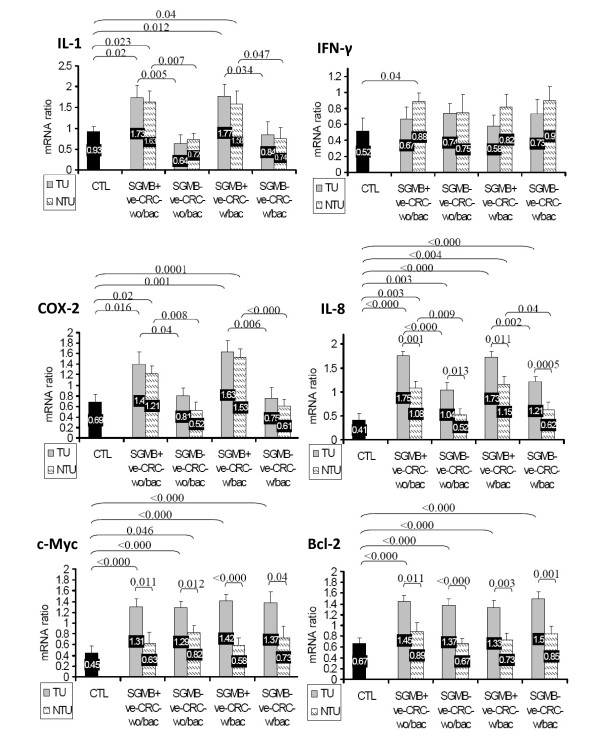
**Normalized mRNA ratio, expressed as mean+ SE, of IL-1, IFN-γ, COX-2, IL-8, c-Myc, and Bcl-2 is shown in TU and NTU tissues of control, SGMB+ve-CRC-wo/bac, SGMB-ve-CRC-wo/bac, SGMB+ve-CRC-w/bac and SGMB-ve-CRC-w/bac**. Significant differences in mean mRNA ratio (P < 0.05) among different groups are shown as demarcation brace lines with corresponding P values whereas insignificant differences are devoid of demarcation and P values.

**Figure 4:**
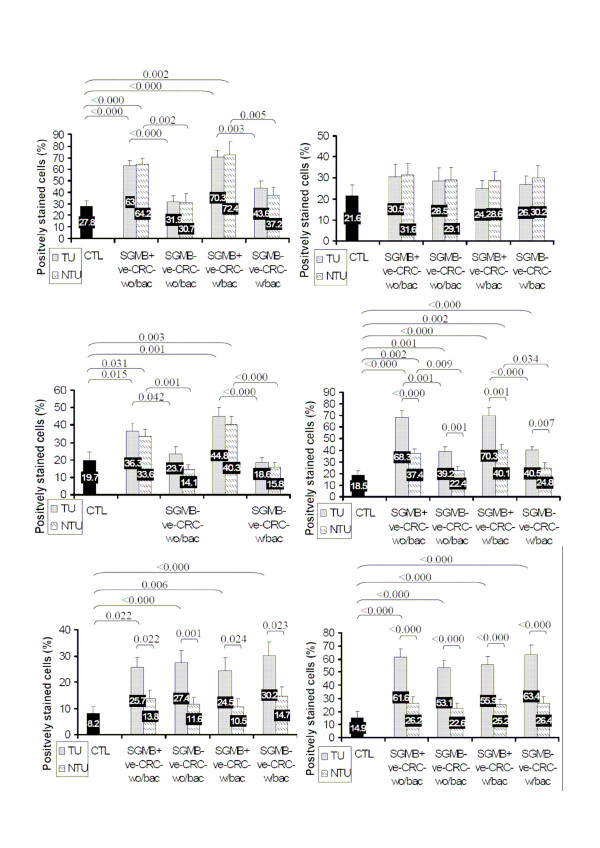
**Mean+SE of positive mRNA staining via ISH assay for IL-1, IFN-γ, COX-2, IL-8, c-Myc, and Bcl-2 is shown in TU and NTU tissues of control, SGMB+ve-CRC-wo/bac, SGMB-ve-CRC-wo/bac, SGMB+ve-CRC-w/bac and SGMB-ve-CRC-w/bac**. Significant differences in mean of positive mRNA staining (P < 0.05) among different groups was shown as demarcation brace lines with corresponding P values whereas insignificant differences were devoid of demarcation and P values.

The comparative results of mRNA level of the studied cytokines and oncogenes among the involved groups of CRC patients and control subjects were very similar in both semi-quantitative real-time RT-PCR, in term of mRNA ratio, and in ISH assay, in term of percentage of the positively stained cells. For c-Myc and Bcl-2, the mRNA expression was higher in TU, but not NTU, of CRC patients than of control group and higher in TU than in NTU in all CRC groups; nevertheless, no significant difference was found in the level of mRNA expression of c-Myc and Bcl-2 regarding the history of bacteremia or the positive detection of SGMB (Figure 3 and 4). For IL-8, mRNA level was higher in TU than in NTU in all groups of CRC patients; IL-8 mRNA was higher in TU and NTU of SGMB+ve groups in both CRC-w/bac and CRC-wo/bac than in SGMB-ve groups; and IL-8 mRNA was higher in TU and NTU of SGMB+ve, but not SGMB-ve, in both CRC-w/bac and CRC-wo/bac than in control group (Figure 3 and 4). On the other hand, the mRNA level of proinflammatory cytokines, IL-1 and COX-2, was at close levels between TU and NTU of all CRC groups; it was significantly higher in TU and NTU of SGMB+ve than in SGMB-ve groups in both CRC-w/bac and CRC-wo/bac; and it was higher in TU and NTU of SGMB+ve, but not SGMB-ve, groups than in control group (Figure 3 and 4). Regarding IFN-γ, the mRNA level was not significantly different between TU and NTU in all CRC groups, between SGMB+ve and SGMB-ve groups in both CRC-w/bac and CRC-wo/bac, and between control and all CRC groups (Figure 3 and 4). Accordingly, the mRNA expression of the studied agents revealed that inflammatory cytokines, IL-1 and COX-2, were strongly associated with SGMB colonization more than transformation process itself while mRNA expression of IFN-γ was not shown to be related significantly with transformation, SGMB, or TU versus NTU. On the contrary, the mRNA expression of oncogenic and anti-apoptotic factors, c-Myc and Bcl-2, was associated with the transformation process but not with SGMB colonization or safe margin tissues, namely NTU. Interestingly, IL-8 proved to be of vital role in the carcinogenesis of colorectal tissues; mRNA expression of IL-8 was associated strongly with SGMB colonization, transformation process, and TU versus NTU tissues. It is worth mentioning that mRNA level in either ISH assay or semi-quantitative RT-PCR was not associated with age and sex of CRC patients or with CRC stage, tumor grade, or location of tumors. However, the mean mRNA ratio and percentage of positively stained cells of Bcl-2 in B1, B2, C1, and C2 stages collectively, 0.84 ± 0.23 and 21.6 ± 6.2% were much lower than in advanced D stage, 1.35 ± 0.12 and 58 ± 7.2, respectively (*P *= 0.01, 0.008 respectively).

### Correlations among mRNA expression of cytokines and with SGMB load

**T**he mRNA ratio of IL-1 was positively correlated with that of COX-2 in TU SGMB+ve-CRC-wo/bac (r = +0.85, *P *= 0.002) and in TU SGMB+ve-CRC-w/bac (r = +0.78, *P *= 0.003). The mRNA ratio of IL-8 was positively correlated with that of COX-2 in TU SGMB+ve-CRC-w/bac (r = +0.86, *P *= 0.001) and, to a lesser extent, in TU SGMB+ve-CRC-wo/bac (r = +0.77, *P *= 0.016).

One of the interesting findings, though might be predicted from the earlier results of the current study, the bacterial load of SGMB, in terms of log_10_CN/g, was positively correlated with mRNA ratio of IL-8 in TU SGMB+ve-CRC-w/bac (r = +0.82.3, *P *= 0.01) and TU SGMB+ve-CRC-wo/bac (r = +0.74, *P *= 0.02), COX-2 in TU SGMB+ve-CRC-w/bac (r = +0.83, *P *= 0.004), and IL-1 in TU SGMB+ve-CRC-w/bac (r = +0.78, *P *= 0.01). Collectively, all positive correlations were observed among IL-1, COX-2, and IL-8 in TU SGMB+ve sections; moreover, the mRNA expression of these three cytokines, rather than other studied agents, was positively correlated with SGMB load in colorectal tissues. This confirmed the earlier findings which pointed out clearly to the remarkable association of IL-8, COX-2 and IL-1 with SGMB colonization of colorectal tumors.

## Discussion

The association of SGMB, previously known as *S. bovis*, with CRC has been reported to be 18 to 62% [6-9]. Hence, Beeching et al proposed that all patients with SGMB bacteremia/endocarditis should be rigorously investigated for the presence of a colonic tumor [25]. However, the association of SGMB with CRC has always been described through the incidence of SGMB bacteremia and/or endocarditis. Little research has been done on elucidating the direct relationship of SGMB with tumor lesions of CRC to confirm or refute, on solid bases, the direct link between CRC and SGMB.

In the current study, the association of SGMB with CRC was confirmed by using biochemical tests, bacteriological isolation of SGMB, and detection of SGMB DNA via PCR and ISH assay at tumor lesions compared to normal colorectal tissues.

*SodA *gene primer- based PCR and S.g.*SodA *probe- based ISH assay were used. Although PCR assay proved to be more sensitive than ISH, both assays yielded very close findings. PCR and ISH assays succeeded in detecting SGMB in colorectal TU and NTU tissues of CRC patients much higher than in control tissues and fairly higher, about two folds, than in the same tissues by using bacteriological approach. In addition, quantitative real-time PCR revealed significantly higher SGMB colonization in the tissues of SGMB+ve CRC patients than in control group, in TU than in NTU tissues, and in SGMB+ve-CRC-w/bac than in SGMB+ve-CRC-wo/bac groups.

The current findings of bacteriological and molecular assays provided invaluable clues and new facts. First, the bacteriological isolation of SGMB without enrichment media was unsuccessful. With enrichment media, SGMB were isolated in half of the colorectal tissues where SGMB were detected by molecular assays. Almost all of the previous studies explored SGMB colonization in colorectum were conducted by using simple bacteriological assays without using proper enrichment media and, above all, they did not use molecular assays targeting SGMB DNA. This explains why previous studies found either no evidence of SGMB colonization at tissues of colorectal carcinomas [26] or low colonization percentage, 11%, which was not significantly different from that of mucosal 5.5%, or stool, 11%, samples [27].

The second essential clue of this study, the positive detection of SGMB was confined only to TU and NTU tissues of CRC patients rather than colorectal tissues of control subjects. And the SGMB load, log_10_CFN/g, was significantly higher in TU than in NTU colorectal tissues. These findings suggest that there might be certain kind of host-bacteria interaction involving both transformed and adjacent histologically-normal tissues, and this host-bacteria interaction could be of etiological role in the carcinogenesis of CRC or of propagating factor in tumor development. Klein et al stated that undefined physical or biochemical factors in CRC tissues may encourage *S. bovis *to be locally carcinogenic [28]. In a study using animal models, the cell wall antigens of *S. bovis *were found to promote premalignant lesions through the increased formation of hyper-neoplastic aberrant colonic crypts [19]. Moreover, *S. bovis *has been shown to be able of causing a longstanding presence in the bowel leading to chronic inflammation in the colon [20]. Accordingly, such chronic bacterial infection/inflammation may, in turn, contribute to cancer development. In addition, it was also suggested that carcinogenesis might issue when bacterial components interfere with cell function [20].

The third essential clue of this study is that the proven colonization of SGMB in TU and NTU tissues of CRC patients could explain why SGMB, more than other dominant intestinal bacteria, can get access through blood vessels into the circulation which in turn leads to bacteremia and/or endocarditis. This interpretation, which was based on the findings of the current study, supports what was suggested by other studies that colonic lesions provide a suitable microenvironment for *S. bovis *resulting in silent tumor-associated infections which become apparent when cancer patients become immunocompromised, like in bacteraemia, or have coincidental cardiac valve lesions, like in endocarditis [16,24,25,29]. In addition, CRC patients with history of bacteremia showed higher frequency of SGMB positive detection as well as higher titer of colonizing SGMB than those without history of bacteremia which provided evidence why some CRC patients develop bacteremia while others do not. Nevertheless, in the current study, SGMB were not detected or isolated in a portion of CRC patients with history of bacteremia. This could be explained that SGMB in some CRC-w/bac patients might be wiped out by antibiotic treatment during bacteremia/endocarditis phase or there might be individual variation among CRC patients in their immunological response to SGMB bacteremia leading to the eradication of SGMB in some patients and the survival of SGMB in others.

The fourth clue of this study, the colonization of SGMB in CRC tumors and safe margin tissues rather than normal tissues indicated that SGMB might possess peculiar adhesive potential to malignant and premalignant colorectal tissues. The findings of other studies might explain the results of the current study. A study found that *S. bovis *common antigen has the ability to facilitate tumor attachment before translocation to the bloodstream [30]. In addition, a histone-like protein A in *S. gallolyticus *was found to bind this bacteria tightly to colon tumor cell lines HCT116 and HT-29 [31]. Moreover, Sillanpaa team found that *S. gallolyticus *has a distinctive ability to bind to all types of collagen proteins in the extra-cellular matrix making *S. gallolyticus *subspecies *gallolyticus *a successful colonizer in both intestinal and cardiac tissues which might explain the association between endocarditis and intestinal lesions caused by this bacterium [32].

The fifth essential outcome of the current study is that both *SodA *gene-specific primers and probe were successfully used in PCR and ISH assays, respectively, for designing highly sensitive detection tools for SGMB colonizing colorectal tissues. Besides, the detection of SGMB DNA showed that how extensively these bacteria are involved in CRC disease and how the active role of SGMB association with CRC have long been underestimated because of the lack of using sensitive molecular methods of detection.

To disclose the underlying nature of SGMB colonization in colorectal tumors, mRNA expression of six cytokines, chemokines and oncogenes were studied via two different assays, ISH and semi-quantitative real-time RT-PCR. Both assays turned out very similar patterns of mRNA expression. Remarkably high mRNA expression of two proinflammatory cytokines, IL-1 and COX-2, was highly associated with SGMB+ve CRC patients equally in both TU and NTU tissues while their level in SGMB-ve CRC patients was low and close to that in control group. This granted a peerless chance to highlight the inflammatory potential of SGMB in colorectal tissues. This finding supports the hypothesis that SGMB exert chronic inflammatory reaction in bowel tissues which might be one of the main factors for promoting or propagating the development of malignant or premalignant lesions. These findings are in agreement with Ellmerich study which showed that in vitro binding of *S. bovis *wall extracted antigens to various cell lines stimulated the production of inflammatory cytokines by those cells [33]. Moreover, it was stated that the production of inflammatory cytokines in response to *S. bovis *including IL-1β, TNF-α, and IL-6 as well as the chemokine IL-8 plays an important role in the normal defense mechanisms of the host [34] resulting in the production of nitric oxide and free radicals such as superoxide, peroxynitrites and hydroxyl radicals [35]. Due to their remarkable mutagenicity, these molecular species might contribute to the neoplastic processes by modifying cellular DNA.

Regarding the angiogenic chemokine, IL-8, it proved to be exclusively essential in CRC, in general, and SGMB-related CRC, in particular. The level of mRNA expression of IL-8 was higher in SGMB+ve than in SGMB-ve groups, higher in TU than in NTU groups, and higher in CRC than in control groups. In other words, IL-8 was shown to be associated with SGMB colonization, transformation process, and local tumorogenesis altogether. This finding renders IL-8 as the most important link between SGMB and carcinogenesis of CRC. Jung et al showed that IL-8 was the most remarkable chemokine expressed by human colon epithelial cells when exposed to invasive strains of *H. pylori *bacteria [36]. Moreover, it was shown that IL-8 production was induced by CRC cells exposed in vitro to Clostridium difficile toxin A [37]. Interestingly, *S. bovis *were previously shown to increase the production of IL-8 and, to a lesser extent, inflammatory cytokines in the colonic mucosa of rats, suggesting direct interactions between *S. bovis *and the colonic mucosal cells via IL-8 [33]. Therefore, IL-8 seems to be strongly related to tumorogenesis when is induced or promoted by microbial-driven inflammation. Because of the fact that IL-1 and COX-2 are products of NFkB activity [38], the current findings regarding the association of IL-1, COX-2, and IL-8 with SGMB+ve CRC confirmed the findings of a previous study conducted by our team where *S. gallolyticus*-seropositive CRC patients were significantly associated with higher mRNA expression of both NFkB and IL-8 [16]. In addition, it was shown in the current study that COX-2 was positively correlated with IL-1 and IL-8; moreover, IL-1, COX-2, and IL-8 were all positively correlated with SGMB load in TU SGMB+ve CRC patients. This provided extra evidence for the integrative role of IL-1, COX-2, and IL-8 in SGMB-related carcinogenesis of CRC. Hence, these cytokines and chemokines most probably act together by perpetuating the SGMB-induced inflammation along with preparing a strong angiogenic environment which is necessary for the development and expansion of tumors.

For the mRNA expression of the oncogene c-Myc and antiapoptotic Bcl-2, they were not associated with SGMB colonization but were associated with CRC transformation. This might imply that SGMB could have nothing to do with inducing oncogenic changes or suppressing cellular apoptosis. Therefore, SGMB more probably act as a propagator factor for premalignant or oncogene-positive tissues to enter the transformation cycle through inflammatory and angiogenic microclimates. However, this has not yet been confirmed and further studies are needed to assess whether SGMB act as carcinogenic or propagator factors for CRC development. On the other hand, the immunological regulator of inflammation, IFN-γ, was not related with SGMB or the transformation process of CRC. This might be attributed to the immunosuppressive environment of tumors that rendered IFN-γ of equivocal effect on SGMB colonization and CRC carcinogenesis.

## Conclusions

Taken together, the current study showed clearly that the colonization of SGMB do exist in TU and NTU tissues of CRC patients, with or without history of bacteremia, while no such colonization was found in colorectal tissues of control subjects suggesting an active role of SGMB in CRC development. Besides, SGMB colonization was heaviest in TU then in NTU and in CRC-w/bac then in CRC-wo/bac highlighting that SGMB load might be the main determinant for developing SGMB bacteremia. Moreover, fecal and mucosal SGMB were shown not to reflect reliably the status of SGMB colonization in colorectal tissues. On the contrary, molecular assays detecting SGMB-specific *SodA *gene, via ISH and PCR, proved to be reliable and much more sensitive than bacteriological approaches. On the other hand, SGMB colonizing colorectum appeared to induce mRNA expression of proinflammatory cytokines, IL-1 and COX-2, as well as angiogenic chemokine, IL-8; these cytokines and chemokines collectively provide bases for promoting/propagating normal or premalignant colorectal tissues into malignant status. It is recommended to conduct further studies to clarify the active role of SGMB in CRC development and pinpoint the molecules in colorectal cells that interact with SGMB leading to such active and, most probably, carcinogenic kind of bacterial colonization.

## Methods

### Population of the study

The involved CRC patients were selected from several gastroenterology centers in the state of Selangor, Malaysia from February 2007 to July 2009. CRC patients were subjected to surgical resection of colorectal cancer and were categorized into two groups, CRC patients with a history of *S. gallolyticus*/bovis bacteremia in the last 2 years, named as CRC-w/bac, and CRC patients without bacteremia/endocarditis, named as CRC/wo-bac. However, antibiotics taken at the time of bacteremia/endocarditis might still be interfering with the results of the study; therefore, the patients of CRC-w/bac group included in this study were those who were treated from bacteremia/endocarditis by intravenous antibiotics, at least 3 months before conducting surgery in order to minimize antibiotic-driven effect on SGMB colonization, if any. Fifty two CRC-wo/bac, 29 men and 23 women aged between 45 to 78 years and 39 CRC-w/bac, 21 men and 18 women, aged between 42 to 81 years with primary colorectal adenocarcinoma were involved in the current study before administration of chemotherapy. CRC patients were examined and their health records were reviewed. The patients who revealed no major illness or gastrointestinal disorder other than CRC and did not receive any antibiotic for the last 3 months, including CRC-w/bac, were only recruited in the current study. On the other hand, 50 age- and sex- matched control subjects were selected from those who were subjected to colonoscopy and their colonic mucosa were shown to be free of gastrointestinal lesions. Moreover, no gastrointestinal or major disease was found in control subjects after conducting a thorough medical examination and after retrieving their medical and surgical history. Colonoscopical biopsies were taken from control subjects for comparing them with excisional biopsies of CRC patients. CRC patients involved in this study were presented at all stages of the disease, B1 to D. Written consents were obtained from the participants. The study was carried out in the scope of Helsinki declaration of ethical principles of medical research and permission was granted from the Ethics Committee of biomedical research.

### Standard bacterial strains

Three different members of SGMB which are genetically close to each other were used as positive standard bacterial strains (PSBS), namely *S. gallolyticus *subspecies *gallolyticus *CIP 105428 (ACM 3611), *S. gallolyticus *subspecies *macedonicus *CIP 105683, and *S. gallolyticus *subspecies *pasteurianus *CIP 105070. On the other hand, closely related taxa and dominant intestinal species of bacteria that are rarely associated with CRC were included as negative standard bacterial strains (NSBS). NSBS included *S. lutetiensis *strain CIP 108103, *S. pyogenes *strain CIP 56.41, *S. equinus *strain CIP 102504, *S. bovis *ACM 3539, *Enterococcus faecalis *CIP 76117, and *Bacteroides fragilis *ATCC 25285. PSBS and NSBS were used to double check the specificity of the already tested [39] manganese-dependent superoxide dismutase (*sodA*) gene-specific primers and *sodA *gene-specific probe at the conditions of the pursued assays of PCR and in situ hybridization (ISH), respectively. All the strains of PSBS were cultured at 37°C on Columbia blood agar (Oxoid Ltd., UK) with 5% horse blood while NSBS bacteria were cultured according to the corresponding media for each species.

### Specimens of colorectal mucosa and tumors

The excisional biopsies of tumor lesions that were taken from CRC patients after surgery and the colonoscopy punch biopsies that were taken from control group were prepared for four different processing pathways: bacteriological isolation of SGMB, DNA extraction for conventional and quantitative real-time PCR targeting DNA of SGMB, mRNA extraction for semi-quantitative real-time RT-PCR of IL-1, IL-8, IFN-γ, COX-2, c-Myc, and Bcl-2, and in situ hybridization (ISH) assay for the detection of *S. gallolyticus SodA *gene, IL-1, IL-8, IFN-γ, COX-2, c-Myc, and Bcl-2 nucleotides. By a histopathologist, biopsies from CRC patients were categorized into 2 groups, tumorous, TU, (from tumor lesion itself) and non-tumorous, NTU, (from histologically tumor-free resection margins). In addition, fecal samples were collected from control subjects as well as from CRC patients preoperatively for assessing and enumerating fecal *S. gallolyticus/bovis *bacteria.

### Isolation of SGMB

SGMB were subjected for isolation from fecal material and colorectal tissues. The isolation and differentiation of target bacteria were based on the protocols of Devriese et al. [12]. For the bacteriological tests, terms like *S. gallolyticus *and *S. bovis *were used rather than SGMB because, by using bacteriological tests, it is difficult to differentiate target bacteria into the molecular-based new terminology used in this study. However, by using API biochemical tests, bacteriologically-proven *S. gallolyticus *were shown to cover SGMB, namely *S. bovis *I and II/2, or *S. gallolyticus gallolyticus *and *S. gallolyticus pasterianus*, while bacteriologically-proven *S. bovis *were shown to represent non-SGMB family, more precisely *S. bovis *biotype II/1 or *S. infantarius *bacteria. For the preparation of specimens, 5 g of feces were 1:5 diluted in brain heart infusion broth (Oxoid, UK) with 5% bovine blood (Oxoid, UK) for 18-24 h at 37°C. For colorectal tissues, 5 g were rinsed thoroughly with shaking for 5 min in 30 ml of PBS for the recovery of mucosally attached target bacteria and PBS was then centrifuged at 3,000 × g for 5 min at 15°C. The pellet was then resuspended in 1 ml of brain heart infusion broth and Columbia agar with 5% bovine blood for 18-24 h at 37°C. On the other hand, for bacteria infiltrating colorectal tissues, the rinsed colorectal tissues (5 g) were washed thoroughly three times with PBS to get rid of any remaining surface bacteria and were then homogenized mechanically and incubated in brain heart infusion broth and Columbia agar with 5% bovine blood for 18-24 h at 37°C. Enriched bacteria were then grown on two selective media, Slantez and Bartley agar (Oxoid, UK) and Edwards agar with 5% bovine blood (Oxoid, UK) at 42°C; plates were incubated in air and supplemented with 5% CO_2 _which increased growth of all strains. *S. gallolyticus *was differentiated from *S. bovis *in selective media by colony morphology and color [12]. In Slantez Bartley medium, dark red colonies were diagnosed as *Enterococcus*, small white colonies as *S. bovis*, and small pale pink colony as *S. gallolyticus*. For Edwards agar, *S. gallolyticus*, unlike *S. bovis*, was grown without brown-black discoloration. In addition, for confirming the identity of target bacteria in terms of *S. bovis *I, II/1, or II/2, API 20, Strep and Rapid ID32 Strep galleries (BioMérieux, La Balme les Grottes, France) were used. Moreover, solution of 6.5% NaCl was also used for salt tolerance [12].

### DNA and RNA extraction of colorectal biopsies

DNA extraction was done according to the instructions of TRI-zol kit (invitrogen, USA) which is a modification of Guanidium Isothiocyanate method. First, a piece of colorectal tissue was put in a tube containing 300 μl Tri-Zol solution. The tissue was minced using the tip of a pointed pincet then vortexed for 1 minute. The tube was left for 5 min at room temperature. Into the tube, 80 μl of chloroform (Merck, Germany) were added. For DNA isolation, the organic phase was then pipetted into a new tube containing 200 μl of protein precipitation solution (Promega, USA) to purify the genomic DNA and the resulting reaction mixture was vigorously vortexed for 20 s. Afterwards, the mixture was incubated in ice for 5 min, and centrifuged at 13,000 × *g *for 3 min at 4°C. The supernatant was carefully transferred into a clean 1.5 ml microcentrifuge tube containing 200 μl of absolute ethanol. The tube was shaken and left for 1 h in 2-8°C. DNA was sedimented by centrifuging in 8,000 × g for 10 min at 4°C and was washed twice using 75% ethanol (Merck, Germany). It was dried for 30 min at room temperature. DNA was suspended using 100 μl DNA rehydration solution (Promega, USA) [40,41]. DNA yield was 10 to 12 μg; the absorbance ratio of 260/280 nm was > 1.8 and DNA quality was assessed further by gel electrophoresis. For RNA isolation, the aqueous part was removed to a Rnase-free 1.5 ml microcentrifuge Tube (Invitrogen, USA) and RNA was precipitated by adding 0.5× volume of 100% room temperature ethanol [42]. Then, RNA was purified by using QIAGEN RNeasy Mini Kit (QIAGEN, Germany); the procedure was conducted according to the protocol outlined in the RNeasy Mini Handbook [43]. Average RNA yield was 4 to 6 μg and absorbance ratio of 260/280 nm was > 1.8. RNA quality was assessed using Agilent 2100 Bioanalyzer with RNA LabChip kit (Agilent, USA).

### DNA extraction of standard bacteria

DNA of the standard bacteria was extracted using Wizard^® ^Genomic DNA Purification Kit with accessory reagents (Promega, USA); the method of extraction was done according to the manufacturer's instructions. The obtained genomic DNA was re-hydrated by adding 100 μl DNA Rehydration solution. Extracted DNA 260/280 nm ratio was > 1.8 and quality was assessed by gel electrophoresis.

### The specificity of SodA gene primers

The primers designed for the current study were used in both conventional and quantitative real-time PCR for the detection of SGMB DNA. The forward primer, **5'**-CAATGACAATTCACCATGA-3', is composed of 19 bases whereas the reverse primer, **5'**-TTGGTGCTTTTCCTTGTG-3', is composed of 18 bases. These primers were already tested by a previous study [39] by which these primers were shown to be highly specific in targeting *SodA *gene for the identification of 23 strains of SGMB including *S. gallolyticus, S. pasteurianus *and *S. macedonicus *strains while these primers were shown to be negative for *S. infantarius*, *S. salivarius*, and *S. equines *[39]. Because these primers were first designed in 2004 and gene bank is expanding progressively every year, the specificity of these primers was rechecked by our team in 2008 and 2009 using Genbank BLAST-Primer program which reconfirmed that these primers are highly specific for SGMB DNA. The nucleotide sequences of *sodA *gene in *S. gallolyticus *subspecies *gallolyticus, S. gallolyticus *subspecies *pasterianus*, and *S. gallolyticus *subspecies *macedonicus *were the only sequences showing complete complimentarity with the tested primers yielding a product of 408 bp, Tm 84.4 C, and GC content 44.3%. The accession numbers of nucleotide sequences that showed complete complimentarity were: FJ617234, FJ617229, FJ617228, FJ042703, FJ151363, FJ151362, FJ151361, FJ151360, FJ151359, FJ151357, FJ151355, DQ232583, DQ232578, DQ232549, AY035715, AY035714, AJ297211, AJ297209, AJ297208, AJ297206, AJ297204, AJ297202, AJ297201, AJ297200, AJ297199, AJ297198, AJ297197, AJ297196, AJ297195, AJ297193, AJ297192, AJ297191, AJ297190, AJ297183, FJ617226, FJ151367, and AY315154. Other strains and species, including *homo sapiens*, were non-complimentary to the tested primers.

In the current study, for more confirmation, the *sodA *gene primers were tested on PSBS that are supposed to be recognized by these primers and NSBS that are not supposed to be recognized by these primers by using conventional PCR assay on DNA extracted from PSBS and NSBS via Wizard^® ^Genomic DNA Purification Kit. Moreover, to simulate DNA extracted from colorectal tissues, *SodA *gene primers were tested on DNA extracted from PSBS or NSBS-inoculated colorectal tissue biopsies of control group via TRI-zol kit, which were named PSBS-tissue and NSBS-tissue, respectively.

### Conventional PCR for bacterial sodA gene

The PCR protocol used was based on a previous study [39] with modifications applied after frequent optimization runs. Two μl, in duplicates, of DNA extracted from PSBS and NSBS, DNA extracted from PSBS-tissue and NSBS-tissue, and DNA extracted from colorectal tissues of CRC and control subjects were added to the PCR master mixture, which consisted of 2 μl of 10× PCR buffer, 0.8 μl of a 10 mM deoxynucleoside triphosphate mixture, 0.7 μl of forward and reverse primers at concentration of 10 pmol/μl, 1.5 μl of 25 mM MgCl_2_, and 0.2 μl of 0.5 U *Taq *DNA polymerase; the remaining volume consisted of distilled water. All the reaction mixtures were obtained from (Promega, USA). The reaction mixture in microcentrifuge tube was amplified in a thermocycler PCR system (PTC-110TM Model, MJ Research, Inc., USA). The PCR protocol consisted of an initial denaturation at 95°C for 5 min with 30 cycles of denaturation at 94°C for 60 s, annealing at 52°C for 30 s, and extension at 72°C for 30 s; and a final extension at 72°C for 8 min. Afterwards, 5 μl of PCR products were electrophoresed on 2% agarose gel using QIAGEN GelPilot DNA Molecular Weight (Qiagen, Germany) as a ladder. PCR products were separated by an electrophoresis system at a constant voltage of 80 V for 50 min and were stained with 0.25 μg/ml ethidium bromide (Sigma, USA). PCR products were photographed under UV transilluminator (Vilber Lourmat, Cedex, France) and the photos were taken using gel documentation system, Bio Rad Gel Doc 2000 Model Imaging System (Bio-Rad, USA). PCR assay yielded an amplicon of 408 bp. Positive control, in duplicate, was included containing DNA from reference *S. gallolyticus *subsp. *gallolyticus *CIP105428, whereas negative control, in duplicate, was included containing all PCR reactive agents except for template DNA.

### Standard curve of real-time PCR

The purified PCR product of interest, 408 bp, obtained from *streptococcus gallolyticus *subsp. *gallolyticus *CIP105428 was ligated into pGEM^®^-T Easy Vector (Promega, USA) which consisted of a mixture of 2× rapid ligation buffer, pGEMTR Easy Vector, purified PCR product, T4 DNA ligase and sterile distilled water. *Escherichia coli *competent cells of strain JM 109 were used to carry pGEM^®^-T Easy Vector (Promega, USA). The plasmid DNA along with PCR product insert was then extracted according to the manufacturer's instructions using Wizard^® ^Plus SV Minipreps (Promega, USA). The initial concentration of the plasmid DNA of interest was 8.3 μg/ml. The plasmid with the correct insert was then 10-fold serially diluted up to 8 dilutions. Since the molecular weight of the plasmid DNA is known, the concentration of these dilutions were then transformed into log copy number (log CN), defined as SGMB load, which was plotted against the threshold cycle (Ct) to generate the standard curve used for the absolute quantification by real-time PCR. Moreover, the plasmid DNA of interest was used as positive control while the negative control was managed to be devoid of template DNA.

### Quantitative real-time PCR for bacterial sodA gene

For quantitative assessment of SGMB load in colorectal tumor biopsies, absolute quantitative real-time PCR was designed using the same *sodA *gene primers used in the conventional PCR with modifications. Real-time PCR amplification reaction was performed with Rotor-GeneTM 3000 (Corbett. Research, Australia) using fluorescent dye SYBR Green (QiagenTM QuantiTect^® ^SYBR Green PCR kit). The reaction was performed in a total volume of 25 μl (2.5 μl of 10× PCR buffer); the composition of the reaction mixture per sample was as follows: 1.8 μl of 25 mM MgCl_2_, 0.7 μl of 10 mM dNTP, 9.5 μl SYBR Green, 1.5 μl of 25 μM reverse and forward primers, 0.125 μl of 5 U/μl Taq DNA polymerase, 5.0 μl DNA template (~16 ng), and the rest was RNase free water. All the reaction mixtures were obtained from Promega (Promega, USA). PCR protocol consisted of an initial denaturation at 95°C for 5 min; 35 cycles of denaturation for 60 s at 94°C, annealing for 30 s at 52°C, and extension for 30 s at 72°C; and a final extension cycle for 8 min at 72°C. The serially diluted standards, positive and negative controls, and samples were all simultaneously assayed, in duplicates, during real-time amplification. At the end of amplification, measurement of the SYBR Green fluorescence was done continuously through conducting the melting curve analysis by slow heating at 0.2°Cs-1 increments from 60 to 99 °C, with continuous fluorescence collection. Accordingly, a melting curve was generated at the end of the PCR amplification for monitoring the specificity of PCR reaction.

### Semi-quantitative real-time RT PCR for IFN-γ, COX-2, IL-1, IL-8, c-Myc, and Bcl-2

One-step RT-PCR was used; 250 ng of RNA were reverse transcribed into cDNA for 10 min at 55°C [44]. Real-time PCR amplification reaction was performed with Rotor-GeneTM 3000 (Corbett. Research, Australia) using fluorescent dye SYBR Green (QiagenTM QuantiTect^® ^SYBR Green PCR kit). Gel electrophoresis and melting curve analysis at 0.2°Cs-1 increments from 60 to 99°C were used for confirming reaction specificity. Human β-actin mRNA was used as an internal control. Amplification was done in a volume of 20 μl for 40 cycles with preliminary 10 min denaturation phase at 95 °C and a final 72°C extension phase for 8 min. The manufacturers and sequences of the used primers together with the corresponding PCR protocols are shown in Table 6. The mRNA expression of target or reference samples was normalized to the mRNA expression of the corresponding human β-actin. The relative expression of the studied mRNA molecules was determined by relating the normalized expression of each target, in duplicates, to the normalized expression of a reference sample to calculate a fold-change value in term of mRNA ratio. Positive controls of the studied targets were supplied by the primers' manufacturers and were all tested to yield consistent positive results whereas the negative controls were those samples prepared without DNA template.

**Table 6:** Details of the primers used for the amplification of IL-1, IFN-γ, COX-2, IL-8, c-Myc, and Bcl-2 with the corresponding targets and PCR conditions.

Target	Primer sequence	PCR conditions	Manufacturer	Reference
β-actin for COX-2, IL-1β, IL-8, and Bcl-2	F-5'-GATGAGATTGGCATGGCTTT-3' R-5'-CACCTTCACCGTTCCAGTTT-3'	15 s 95°C, 20 s 60°C, 60 s 72°C	Maximbio, USA	[48]
COX-2	F-5'-TCCTATTATACTAGAGCCCTTCCT-3' R-5'-TTCCACAATCTCATTTGAATCAGG-3'.	15 s 95°C, 20 s 60°C, 30 s 72°C	Maximbio, USA	[48]
IL-1β	F, 5'-TCCAGGGACAGGATATGGAG-3', R, 5'-TCTTTCAACACGCAGGACAG-3' (133 bp);	20 s 95°C, 60 s 60°C, 30 s 72 s	Promega,	[49]
IL-8	F-TAGCAAAATTGAGGCCAAGG R-GGACTTGTGGATCCTGGCTA	20 s 95°C, 60 s 60°C, 30 s 72 s	DNASTAR, USA	[50]
Bcl-2	F-5'acatcgccctgtggatgact3' R-5'gggccgtacagttccacaaa3	95°C 3 min, 40 cycles 95°C 30 s, 58°C 30 s, and 73°C 30 s	Promega, USA	[51]
β-actin for IFN-γ and c-Myc	F-5'-GATGGCCACGGCTGCTT-3' R-5'-ACCCTCATTGCCAATGGT-3'	20 s 95°C, annealing/extension 60 s 60°C	Maximbio, USA	[52]
IFN-γ	F-5'-CAGCTCTGCATCGTTTTGGG-3' R-5'-GTTCCATTATCCGCTACATCTGAA-3'	20 s 95°C, annealing/extension 60 s 60°C	DNASTAR, USA	[52]
c-Myc	F-TCAAGAGGTGCCACGTCTCC R-TCTTGGCAGCAGGATAGTCCTT	30 s 95°C, 70 s 60°C (annealing/extension).	Maximbio, USA	[50]

### Probes used for in situ hybridization assay

For the detection of *SodA *gene DNA via ISH assay, *SodA *gene-specific biotinylated probe was designed according to the nucleotide sequence accession no. AJ297183 by using megablast and discontiguous megablast algorithms for alignment similarity (GenBank, BLAST). The probe size is 435 bp of linear DNA targeted for *sodA *gene of superoxide dismutase of *S. gallolyticus *subspecies *gallolyticus *(strain CIP 105428T, ACM 3611). This probe was named as S.g-*sodA *probe. It was synthesized and biotinylated by (Promega laboratories, USA). S.g-*sodA *probe was designed to be highly specific for *S. gallolyticus *subspecis *gallolyticus*, *S. gallolyticus *subspecis *pasteurianus*, and *S. gallolyticus *subspecies *macedonicus *while it was designed to be incompatible with other related taxa of bacteria, or other organisms' genomes including *Homo sapiens. *This design is necessary to avoid mispriming with DNA of other bacteria or of huamn colroectal tissues. For more confimration, the specificity of S.g-sodA probe was assessed by our team by using ISH assay on duplicates of three strains of PSBS-tissue and six srtains of NSBS-tissue sections at identical conditions to these used for test sections. The strains of PSBS-tissues were CIP 105428, CIP 105683, and CIP 105070 while the strains of NSBS-tissues were CIP 108103, CIP 56.41, CIP 102504, ACM 3539, CIP 76117, and ATCC 25285.

For the mRNA detection of targeted cytokines and oncogenes via ISH assay, biotinylated long cDNA probes were used including human mRNA of IFN-γ (MaxHyb Probe, human IFN-γ, PB-60088, probe size 423 bp, GenBank Accession No: NM_000619, Maximbio, USA), COX-2 (MaxHyb Probe, human COX2, PB-60161, probe size 278 bp, GenBank Accession No: BC013734, Maximbio, USA), Bcl-2 (MaxHyb Probe, human Bcl-2, PB-60010, probe size 235 bp, GenBank Accession No: M14745, maximbio, USA), c-Myc (MaxHyb Probe, human c-Myc, PB-60052, probe size 1181, GenBank Accession No: V00568, Maximbio, USA), IL-1β (MaxHyb Probe, human IL-1β, PB-60089, probe size 196 bp, Genbank accession no. NM_000576, Maximbio, USA), and IL-8 ((MaxHyb Probe, human Interleukin 8, PB-60092, probe size 299 bp, Genbank accession no. NM_000584, Maximbio, USA).

### In situ hybridization for SodA gene, IFN-γ, COX-2, IL-1, IL-8, c-Myc, and Bcl-2

Proper histopathological processing necessitated several steps to be pursued by the pathologist to minimize the fixation-associated loss of mRNA including minimal prefixation time, up to one hour, using cold 4% paraformaldehyde, cold fixation at 4°C, and short duration of fixation, up to 5 h [45]. Paraffin-embedded sections were processed for ISH assay within 3 days before the examination because it was reported that no remarkable loss of nucleic acids is found in the first three days of fixation and paraffin embedding [46]. Histopathological paraffin blocks, whether for CRC patients or control subjects, were processed into sections of 4 um thickness. From both TU and NTU tissues, numerous histopathological sections were made for each CRC patient. Hematoxylin and Eosin slides were prepared by the pathologist to confirm the histopathological diagnosis and the grading of CRC. One negative control tissue section was used at every run of ISH by using diluent buffer instead of probes. In addition, a consistently and strongly positive section was used as positive control along with one endogenous positive probe control. The procedure was pursued according to "DNA probe Hybridization/Detection System - in situ Kit" (Maximbio, USA) with some modifications according to Abdulamir et al [16].

### Staining analysis

**Figure 5:**
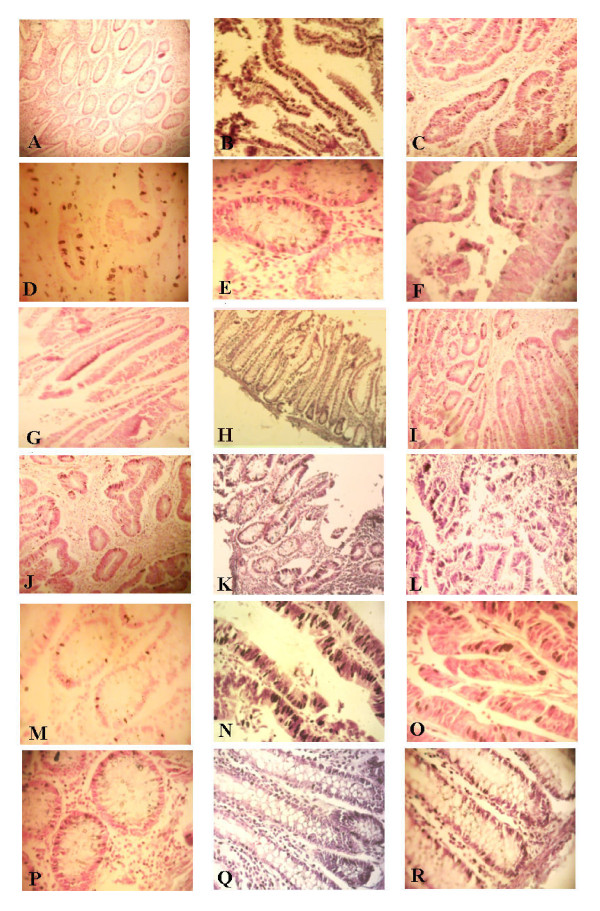
**Examples of ISH staining of mRNA of IL-1, IFN-γ, COX-2, IL-8, c-Myc, and Bcl-2**. Cells stained positive are shown with dark color of NBT/BCIP in the nuclei of glandular cells as well as stromal cells. mRNA expression of IL-1 (A-C) at X100, IFN-γ (D-F) at 400X, COX-2 (G-I) at X100, and IL-8 (J-L) at 100X, is shown in control, TU SGMB+ve-CRCw/bac, and TU SGMB-ve-CRC-w/bac, respectively. mRNA expression of c-Myc (M-O) at X400 and Bcl-2 (P-R) at X400 is shown in control, TU SGMB-ve-CRC-wo/bac, and NTU SGMB-ve-CRC-wo/bac.

**Figure 6:**
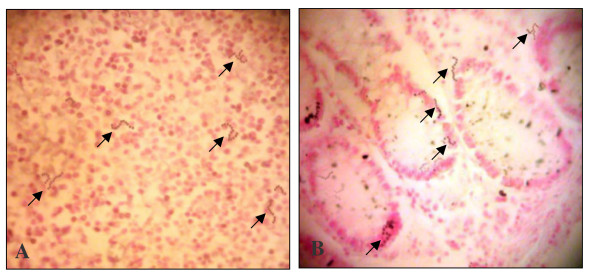
**Examples of ISH staining of SGMB DNA at 400X in colorectal tissues of (A) TU SGMB+ve-CRC-w/bac and (B) NTU SGMB+ve-CRC-wo/bac**. SGMB DNA staining was shown as chains of stained tiny dark spots which are pointed by black arrows. No distinctive pattern of distribution of the positively stained SGMB was seen throughout stained sections.

For mRNA expression of the studied cytokines, chemokines, and oncogenes, the percentage of stained glandular and stromal cells with blue/black nuclear staining was calculated out of total cells in 5 high power fields. Regarding IFN-γ, IL-1, IL-8, COX-2, c-Myc, and Bcl-2, many scoring systems, sometimes contradicting, were found; however, to keep solid comparative basis among the studied groups and to compare ISH results with that of the semi-quantitative real-time RT-PCR, no certain scoring system was used. Instead, the percentage of the positively stained cells out of total cells was used; examples of ISH mRNA staining are shown in Figure 5. On the other hand, stained SGMB DNA appeared as chains of dark tiny spots scattered throughout the examined sections representing the colonizing SGMB; examples of ISH staining of SGMB DNA are shown in Figure 6. The interpretation of ISH staining by S.g-*sodA *probe was assessed qualitatively. Since the presence of SGMB DNA in colorectal tissues is considered abnormal, any positive detection of SGMB DNA was considered positive.

### Statistical analysis

Statistical analysis was conducted using SPSS software version 14.2.1 and MS EXCEL 2007. The normality of quantitative continuous data was tested by Kolmogorov-Smirnov test. Data in the current study were found to be normally distributed; therefore, quantitative data were expressed in mean ± SEM and, therefore, parametric tests were used. Depending on F-test for variance, univariate and multivariate student *t*-test were used for measuring the significance of difference between two means. Categorized qualitative (frequency) data were treated by chi square for independence or Fisher's exact test, when needed, for measuring the significance of association among different studied groups. Moreover, Pearson correlation coefficient (r) was used along with ANOVA test for assessing the significance of correlation. For SGMB cell count, or SGMB load, in colorectal tissues, it was transformed into logarithmic values in terms of log_10 _copy number per gram (log_10 _CN/g). The mean log_10 _CN/g was calculated by averaging the individual log_10 _CN/g values [47]. 95% confidence interval (CI) of mean log_10 _CN/g was used to express uncertainty measurements of bacterial counts and to measure the significance of difference among different groups. *P *values < 0.05 were considered significant.

## List of abbreviations

CN: copy number; CRC: colorectal cancer; Ct: threshold cycle; CRC-w/bac: colorectal cancer with bacteremia; CRC-wo/bac: colorectal cancer without bacteremia; ISH: in situ hybridization; NSBS: negative standard bacterial; PSBS: positive standard bacterial; SGMB: streptococcus gallolyticus member bacteria; SGMB+ve: SGMB positive; SGMB-ve: SGMB negative; sodA: superoxide dismutase A; TU: tumorous; NTU: non-tumorous.

## Competing interests

The authors of the current manuscript confirm that there is no any kind of competing interests.

## Authors' contributions

All authors read and approved the final manuscript. AS and RR did sampling of specimens, RR performed the bacteriological assays and AS performed the molecular assays. F and AS performed tissue staining and statistical design.

## References

[B1] Triantafillidis JK Nasioulas G Kosmidis PA Colorectal cancer and inflammatory bowel disease: epidemiology, risk factors, mechanisms of carcinogenesis and prevention strategies Anticancer Res 2009 29 7 2727 2737 19596953

[B2] Ablashi DV Chatlynne LG Whitman JE Jr Cesarman E Spectrum of Kaposi's sarcoma-associated herpesvirus, or human herpesvirus 8, diseases Clin Microbiol Rev 2002 15 3 439 464 10.1128/CMR.15.3.439-464.2002 12097251 PMC118087

[B3] Boshoff C Weiss R AIDS-related malignancies Nat Rev Cancer 2002 2 5 373 382 10.1038/nrc797 12044013

[B4] Parsonnet J Bacterial infection as a cause of cancer Environ Health Perspect 1995 103 Suppl 8 263 268 10.2307/3432323 8741796 PMC1518971

[B5] Malfertheiner P Sipponen P Naumann M Moayyedi P Megraud F Xiao SD Sugano K Nyren O Helicobacter pylori eradication has the potential to prevent gastric cancer: a state-of-the-art critique Am J Gastroenterol 2005 100 9 2100 2115 10.1111/j.1572-0241.2005.41688.x 16128957

[B6] Wilson WR Thompson RL Wilkowske CJ Washington JA Giuliani ER Geraci JE Short-term therapy for streptococcal infective endocarditis. Combined intramuscular administration of penicillin and streptomycin JAMA 1981 245 4 360 363 10.1001/jama.245.4.360 7452862

[B7] Reynolds JG Silva E McCormack WM Association of Streptococcus bovis bacteremia with bowel disease J Clin Microbiol 1983 17 4 696 697 6853693 10.1128/jcm.17.4.696-697.1983 PMC272719

[B8] Leport C Bure A Leport J Vilde JL Incidence of colonic lesions in Streptococcus bovis and enterococcal endocarditis Lancet 1987 1 8535 748 10.1016/S0140-6736(87)90391-6 2882164

[B9] Zarkin BA Lillemoe KD Cameron JL Effron PN Magnuson TH Pitt HA The triad of Streptococcus bovis bacteremia, colonic pathology, and liver disease Ann Surg 1990 211 6 786 791 discussion 791-782 10.1097/00000658-199006000-00019 2357141 PMC1358139

[B10] Murray PR Baron EJ Manual of clinical microbiology 2007 ASM Press, Washington, D.C, USA

[B11] Osawa R Fujisawa T Sly LI Streptococcus gallolyticus sp. nov.: gallate degrading organisms formerly assigned to Streptococcus bovis Syst Appl Microbiol 1995 18 74 78

[B12] Devriese LA Vandamme P Pot B Vanrobaeys M Kersters K Haesebrouck F Differentiation between Streptococcus gallolyticus strains of human clinical and veterinary origins and Streptococcus bovis strains from the intestinal tracts of ruminants J Clin Microbiol 1998 36 12 3520 3523 9817865 10.1128/jcm.36.12.3520-3523.1998 PMC105232

[B13] Schlegel L Grimont F Ageron E Grimont PA Bouvet A Reappraisal of the taxonomy of the Streptococcus bovis/Streptococcus equinus complex and related species: description of Streptococcus gallolyticus subsp. gallolyticus subsp. nov., S. gallolyticus subsp. macedonicus subsp. nov. and S. gallolyticus subsp. pasteurianus subsp. nov Int J Syst Evol Microbiol 2003 53 Pt 3 631 645 10.1099/ijs.0.02361-0 12807180

[B14] Lee RA Woo PC To AP Lau SK Wong SS Yuen KY Geographical difference of disease association in Streptococcus bovis bacteraemia J Med Microbiol 2003 52 903 908 10.1099/jmm.0.05199-0 12972586

[B15] Jean SS Teng LJ Hsueh PR Ho SW Luh KT Bacteremic Streptococcus bovis infections at a university hospital, 1992-2001 J Formos Med Assoc 2004 103 118 123 15083242

[B16] Abdulamir AS Hafidh RR Mahdi LK Al-jeboori T Abubaker F Investigation into the controversial association of Streptococcus gallolyticus with colorectal cancer and adenoma BMC Cancer 2009 9 403 10.1186/1471-2407-9-403 19925668 PMC2785837

[B17] Beck M Frodl R Funke G Comprehensive study of strains previously designated Streptococcus bovis consecutively isolated from human blood cultures and emended description of Streptococcus gallolyticus and Streptococcus infantarius subsp. coli J Clin Microbiol 2008 46 2966 2972 10.1128/JCM.00078-08 18614655 PMC2546750

[B18] Corredoira J Alonso MP Coira A Casariego E Arias C Alonso D Pita J Rodriguez A Lopez MJ Varela J Characteristics of Streptococcus bovis endocarditis and its differences with Streptococcus viridans endocarditis Eur J Clin Microbiol Infect Dis 2008 27 285 291 10.1007/s10096-007-0441-y 18183440

[B19] Ellmerich S Scholler M Duranton B Gosse F Galluser M Klein JP Raul F Promotion of intestinal carcinogenesis by Streptococcus bovis Carcinogenesis 2000 21 4 753 756 10.1093/carcin/21.4.753 10753212

[B20] Biarc J Nguyen IS Pini A Gosse F Richert S Thierse D Van Dorsselaer A Leize-Wagner E Raul F Klein JP Scholler-Guinard M Carcinogenic properties of proteins with pro-inflammatory activity from Streptococcus infantarius (formerly S.bovis) Carcinogenesis 2004 25 8 1477 1484 10.1093/carcin/bgh091 14742316

[B21] Wentling GK Metzger PP Dozois EJ Chua HK Krishna M Unusual bacterial infections and colorectal carcinoma--Streptococcus bovis and Clostridium septicum: report of three cases Dis Colon Rectum 2006 49 8 1223 1227 10.1007/s10350-006-0576-4 16845563

[B22] Rochlitz CF Herrmann R de Kant E Overexpression and amplification of c-myc during progression of human colorectal cancer Oncology 1996 53 6 448 454 10.1159/000227619 8960139

[B23] Manne U Myers RB Moron C Poczatek RB Dillard S Weiss H Brown D Srivastava S Grizzle WE Prognostic significance of Bcl-2 expression and p53 nuclear accumulation in colorectal adenocarcinoma Int J Cancer 1997 74 3 346 358 10.1002/(SICI)1097-0215(19970620)74:3<346::AID-IJC19>3.0.CO;2-9 9221816

[B24] Burns CA McCaughey R Lauter CB The association of Streptococcus bovis fecal carriage and colon neoplasia: possible relationship with polyps and their premalignant potential Am J Gastroenterol 1985 80 1 42 46 3966453

[B25] Beeching NJ Christmas TI Ellis-Pegler RB Nicholson GI Streptococcus bovis bacteraemia requires rigorous exclusion of colonic neoplasia and endocarditis Q J Med 1985 56 220 439 450 4048386

[B26] Norfleet RG Mitchell PD Streptococcus bovis does not selectively colonise colorectal cancer and rectal polyps J Clin Gastroenterol 1993 17 25 28 10.1097/00004836-199307000-00008 8409294

[B27] Potter MA Cunliffe NA Smith M Miles RS Flapan AD Dunlop MG A prospective controlled study of the association of Streptococcus bovis with colorectal carcinoma J Clin Pathol 1998 51 6 473 474 10.1136/jcp.51.6.473 9771449 PMC500753

[B28] Klein RS Recco RA Catalano MT Edberg SC Casey JI Steigbigel NH Association of Streptococcus bovis with carcinoma of the colon N Engl J Med 1977 297 15 800 802 10.1056/NEJM197710132971503 408687

[B29] Tjalsma H Scholler-Guinard M Lasonder E Ruers TJ Willems HL Swinkels DW Profiling the humoral immune response in colon cancer patients: diagnostic antigens from Streptococcus bovis Int J Cancer 2006 119 9 2127 2135 10.1002/ijc.22116 16841330

[B30] Kaplan MH Chmel H Stephens A Hsieh HC Tenenbaum MJ Rothenberg IR Joachim GR Humoral reactions in human endocarditis due to Streptococcus bovis: evidence for a common S bovis antigen J Infect Dis 1983 148 2 266 274 6886490 10.1093/infdis/148.2.266

[B31] Boleij A Schaeps RM de Kleijn S Hermans PW Glaser P Pancholi V Swinkels DW Tjalsma H Surface-exposed histone-like protein a modulates adherence of Streptococcus gallolyticus to colon adenocarcinoma cells Infect Immun 2009 77 12 5519 5527 10.1128/IAI.00384-09 19752027 PMC2786452

[B32] Sillanpaa J Nallapareddy SR Singh KV Ferraro MJ Murray BE Adherence characteristics of endocarditis-derived Streptococcus gallolyticus ssp. gallolyticus (Streptococcus bovis biotype I) isolates to host extracellular matrix proteins FEMS Microbiol Lett 2008 289 1 104 109 10.1111/j.1574-6968.2008.01378.x 19054100

[B33] Ellmerich S Djouder N Scholler M Klein JP Production of cytokines by monocytes, epithelial and endothelial cells activated by Streptococcus bovis Cytokine 2000 12 1 26 31 10.1006/cyto.1999.0521 10623439

[B34] Janeway C Immunobiology: the immune system in health and disease 2005 Garland Science, New York, USA

[B35] Ohshima H Bartsch H Chronic infections and inflammatory processes as cancer risk factors: possible role of nitric oxide in carcinogenesis Mutat Res 1994 305 2 253 264 7510036 10.1016/0027-5107(94)90245-3

[B36] Jung HC Eckmann L Yang SK Panja A Fierer J Morzycka-Wroblewska E Kagnoff MF A distinct array of proinflammatory cytokines is expressed in human colon epithelial cells in response to bacterial invasion J Clin Invest 1995 95 1 55 65 10.1172/JCI117676 7814646 PMC295369

[B37] Mahida YR Makh S Hyde S Gray T Borriello SP Effect of Clostridium difficile toxin A on human intestinal epithelial cells: induction of interleukin 8 production and apoptosis after cell detachment Gut 1996 38 3 337 347 10.1136/gut.38.3.337 8675084 PMC1383060

[B38] Spehlmann ME Eckmann L Nuclear factor-kappa B in intestinal protection and destruction Curr Opin Gastroenterol 2009 25 2 92 99 10.1097/MOG.0b013e328324f857 19528876

[B39] Sasaki E Osawa R Nishitani Y Whiley RA Development of a diagnostic PCR assay targeting the Mn-dependent superoxide dismutase gene (sodA) for identification of Streptococcus gallolyticus J Clin Microbiol 2004 42 3 1360 1362 10.1128/JCM.42.3.1360-1362.2004 15004119 PMC356867

[B40] Chomczynski P Mackey K Short technical report. Modification of the TRIZOL reagent procedure for isolation of RNA from Polysaccharide-and proteoglycan-rich sources Biotechniques 1995 19 942 5 8747660

[B41] TRIzol^® ^Reagent and TRIzol^® ^LS Reagent. DNA Isolation using TRIzol 16 20 http://vre.upei.ca/mhl/system/files/Trizol.pdf

[B42] Chomczynski P Bowser-Finn R Sabatini L A reagent for the single-step isolation of viral RNA from human serum and biopsy samples J NIH Res 1994 6 83

[B43] "RNeasy Mini Handbook; RNeasy Mini Protocol for RNA Clean-up," 79 81 http://www.qiagen.com/literature/rnalit.asp#mini

[B44] Schostak M Krause H Miller K Schrader M Weikert S Christoph F Kempkensteffen C Kollermann J Quantitative real-time RT-PCR of CD24 mRNA in the detection of prostate cancer BMC Urol 2006 6 7 10.1186/1471-2490-6-7 16539730 PMC1435920

[B45] Srinivasan M Sedmak D Jewell S Effect of fixatives and tissue processing on the content and integrity of nucleic acids Am J Pathol 2002 161 6 1961 1971 12466110 10.1016/S0002-9440(10)64472-0 PMC1850907

[B46] Lisowski AR English ML Opsahl AC Bunch RT Blomme EA Effect of the storage period of paraffin sections on the detection of mRNAs by in situ hybridization J Histochem Cytochem 2001 49 7 927 928 11410620 10.1177/002215540104900716

[B47] Drake D Assessment of antimicrobial activity against biofilms Methods Enzymol 2001 337 385 389 10.1016/S0076-6879(01)37027-1 11398444

[B48] Cui X Yang SC Sharma S Heuze-Vourc'h N Dubinett SM IL-4 regulates COX-2 and PGE2 production in human non-small cell lung cancer Biochem Biophys Res Commun 2006 343 4 995 1001 10.1016/j.bbrc.2006.03.073 16574063

[B49] Welsh N Cnop M Kharroubi I Bugliani M Lupi R Marchetti P Eizirik DL Is there a role for locally produced interleukin-1 in the deleterious effects of high glucose or the type 2 diabetes milieu to human pancreatic islets? Diabetes 2005 54 11 3238 3244 10.2337/diabetes.54.11.3238 16249450

[B50] McLachlan JL Sloan AJ Smith AJ Landini G Cooper PR S100 and cytokine expression in caries Infect Immun 2004 72 7 4102 4108 10.1128/IAI.72.7.4102-4108.2004 15213155 PMC427449

[B51] Steger K Wilhelm J Konrad L Stalf T Greb R Diemer T Kliesch S Bergmann M Weidner W Both protamine-1 to protamine-2 mRNA ratio and Bcl2 mRNA content in testicular spermatids and ejaculated spermatozoa discriminate between fertile and infertile men Hum Reprod 2008 23 1 11 16 10.1093/humrep/dem363 18003625

[B52] Zhang Y Wang YL Liu YW Li Q Yuan YH Niu WY Sun LY Zhu ZJ Shen ZY Han RF Change of peripheral blood mononuclear cells IFN-gamma, IL-10, and TGF-beta1 mRNA expression levels with active human cytomegalovirus infection in orthotopic liver transplantation Transplant Proc 2009 41 5 1767 1769 10.1016/j.transproceed.2009.03.064 19545724

